# Orally administered recombinant *Lactobacillus* expressing African swine fever virus antigens that induced immunity responses

**DOI:** 10.3389/fmicb.2022.1103327

**Published:** 2023-01-09

**Authors:** Hongliang Zhang, Saisai Zhao, Haojie Zhang, Yu Shen, Peijun Zhang, Hu Shan, Xiulei Cai

**Affiliations:** ^1^College of Veterinary Medicine, Qingdao Agricultural University, Qingdao, Shandong, China; ^2^College of Animal Science and Technology, Shandong Agricultural University, Tai’an, Shandong, China

**Keywords:** African swine fever virus, enterotoxin B subunit, *Lactococcus lactis*, recombinant expression, oral immunization, immunogenicity evaluation

## Abstract

African swine fever (ASF) is a highly contagious, acute, febrile disease caused by the African swine fever virus (ASFV), with morbidity and mortality rates approaching 100% in domestic and wild swine, resulting in massive economic losses to the pig industry worldwide. This study aimed to express the p30, p54, and p72 proteins encoded by ASFV *in vitro* using the *Lactobacillus lactis* (*L. lactis*) expression system. Here, six new functional recombinant *L. lactis* were constructed, and the expression of the p30 protein, p54 protein, p72 protein, p30-LTB (heat-labile enterotoxin B, LTB) fusion protein, p54-LTB fusion protein, and the p72-LTB fusion protein was successfully detected by Western blot analysis. Following oral immunization of rabbits with recombinant *L. lactis*, serum IgG, intestinal mucosal sIgA, cytokines (IL-4 and INF-γ), and splenocyte viability were higher than in the control group *via* ELISA. Notably, without the LTB adjuvant group, humoral and Th1 cellular immunity were promoted, whereas, with the LTB adjuvant group, local mucosal immunity, humoral immunity, and Th2 cellular immunity were promoted, providing new insights into the design and development of an ASFV subunit vaccine.

## 1. Introduction

African swine fever (ASF) is a devastating infection that causes high fever, loss of appetite, and punctate bleeding of the skin and internal organs, with a lethality rate of up to 100% (Pietschmann et al., 2015; Gaudreault et al., 2020). ASF has been identified in sub-Saharan African nations since it was originally discovered in Kenya in the 1920s. In 2007, the disease became active in Georgia’s Caucasus region, from which ASFV gradually spread to neighboring countries such as Armenia, Azerbaijan, Russia, and Belarus, affecting both domestic and wild boars (Rowlands et al., 2008; Costard et al., 2009). A highly virulent genotype II strain of ASFV was introduced into China in 2018, causing unprecedented losses in the pig farming industry (Zhou et al., 2018; Wen et al., 2019; Zhao et al., 2019).

Currently, effective vaccinations and other treatments are not available. So far, biosecurity precautions are the primary means of preventing ASF, but it is impossible to guard against it. Although there have been significant advances in laboratory research, the complexity of the ASFV genome, a large number of encoded proteins and the many unresolved aspects of the virus replication process make it impossible to fill the gap left by a commercially available vaccine. (Sánchez-Vizcaíno et al., 2015; Simões et al., 2019). Many studies provided critical insights into subunit vaccines with safety advantages as a current research hotspot, focusing on structural proteins p30, p54, p72, pp62, EP153R, D117L, and CD2v by incorporating multitarget cocktails or immunization with specific ASFV antigen targets (Ruiz-Gonzalvo et al., 1996; Hernáez et al., 2004; Gaudreault and Richt, 2019; Wu et al., 2020). Specifically, previous research has demonstrated that the p54 and p72 proteins inhibit virus adsorption, p72 and p30 could activate the cytotoxic T lymphocyte (CTL) response, and the p30 protein inhibits virus internalization (Gómez-Puertas et al., 1996; Neilan et al., 2004). However, several subunit vaccine strategies (p30, p54, and p72) used as antigens have been demonstrated with limited progress and inconsistent results, and antigen alone was not promising in protecting animals against virus challenge (Gómez-Puertas et al., 1998). Taken together, the proposed design of vaccine, in the future, is better to address these questions of how to induce the body to produce more effective neutralizing antibodies, how to identify more protective antigens in ASFV, and how to screen for efficient adjuvants to enhance the immune efficacy of subunit vaccines. This work designed a subunit vaccine of p30, p54, and p72 regarded as target proteins for an adaptable means to protect domestic and wild swine from devastating ASF outbreaks, which are described to be the most satisfactory antigenic proteins that cause humoral immune responses during infection.

*Lactococcus* is widely used as a host bacterium to express foreign proteins. It has obvious advantages as a carrier by regulating intestinal flora for maintaining intestinal stability and improving immunity (Bolotin et al., 2001). It also has specific biological activities to stimulate mucosal immunity, such as immune modification and adjuvant activity. Heat-labile enterotoxin B (LTB) subunit can directly bind to antigens and reach immature antigen-presenting cells to produce local and systemic immune responses by stimulating the body. When combined with an antigen, it can induce a strong antitoxin response that enhances the body’s immune response (Duan et al., 2019). Based on the above studies, the authors first proposed using *L. lactis* as the host bacteria and LTB as the adjuvant for the attempt of an ASF subunit vaccine.

A superior formulation for immunoprophylaxis against ASFV infection was to produce a novel and effective oral vaccine based on *L. lactis*. The p30, p54, and p72 optimized structural domains of the ASFV proteins were used as immunogens, and pMG36e was used as an expression delivery vector to successfully create the genetically modified recombinant plasmids pMG36e-*p30*-His, pMG36e-*p54*-His, pMG36e-*p72*-His, pMG36e-*p30*-LTB-His, pMG36e-*p54*-LTB-His, and pMG36e-*p72*-LTB-His. The six plasmids were subsequently electrotransformed into *L. lactis* MG1363, which can colonize the rabbit’s intestinal mucosa and express the p30/p54/p72 proteins and p30/p54/p72-LTB fusion proteins. The results of IL-4 in the serum and sIgA in the small intestine *via* ELISA proved that the p30/p54/p72 proteins, as well as the p30/p54/p72-LTB fusion proteins, all, produced specific antibodies in the gut, which were also produced as sIgA antibodies.

To summarize, preliminary animal experiments in rabbits showed that *Lactobacillus* improves mucosal, humoral, and cellular immunity against ASFV. This study is significant and unique in that it demonstrated support for the development of an oral subunit vaccine for ASFV using *Lactobacillus*.

## 2. Materials and methods

### 2.1. Animals and ethics statement

The rabbits used in this study were purchased from KangDa Biotechnology Co., Ltd. (Qingdao, China). All animal experimental procedures were evaluated and approved by the Institutional Animal Care and Use Committee (IACUC) of the Qingdao Haihua Bio-pharmaceutical Technology Co., Ltd. (Haihua).

### 2.2. Construction of cloning plasmids

The optimized gene fragment sequence (in the supplementary document) in our study, three fragments including the *p30* gene (GenBank No. M96354.1), which encodes amino acids 46 to 251, the *p54* gene (GenBank No. MZ812353.1), which encodes amino acids 18 to 203, and the *p72* gene (GenBank No. MN886930.1), which encodes amino acids 213 to 358, were used as the target gene fragments, intending that specific antigens play a vital role in vaccines to improve high antigenicity and hydrophilicity of antigens, and the base sequences were obtained from NCBI, as well as the LTB gene (GenBank Nos. AF359362.1) and the His-tag gene sequences, which were inserted into downstream of the target genes, and all of the genes were cloned into the pMG36e vector (*Lactobacillus* expression plasmid vector, derived from our laboratory). Six recombinant plasmids, including pMG36e-*p30*/*p54*/*p72*-His and pMG36e-*p30*/*p54*/*p72*-LTB-His, were synthesized by Zhongmei Taihe Biotechnology Co., Ltd. (Beijing, China) and sequenced by Paisenor Gene Biotechnology Co., Ltd. (Qingdao, China).

### 2.3. Construction of recombinant *Lactobacillus lactis* strains

For the construction of the recombinant *Lactococcus lactis* MG1363, the MG1363 competent cells were prepared as follow. The MG1363 stored in our laboratory at –80°C was scribed on a GM17 plate and incubated at 30°C for 36 h, Then, single colony was inoculated in 2 ml of GM17 liquid medium and incubated at 30°C for 24 h, and then inoculate into 30 ml SGM17G hypertonic medium according to 5% inoculation ratio and cultured until OD_600_ = 0.8. Before centrifuging at 5,000 g for 10 min, the bacterial solution was incubated in an ice bath for 10 min at 4°C, and the supernatant was discarded. The precipitate was resuspended by 10 ml pre-chilled Wash Buffer with 10% glycerin containing 0.5 M sucrose by centrifugation at 5,000 g for 10 min at 4°C, discard the supernatant, and repeat twice. Ultimately, the cells were resuspended as competent cells by 400 μl pre-cooled washing solution.

The six recombinant plasmids were electroporated into MG1363 competent cells using an electrotransformer (2,200 V, 200 Ω, 2 msec). The recombinant plasmids pMG36e-*p30*/*p54*/*p72*-His and pMG36e-*p30*/*p54*/*p72*-LTB-His were identified by PCR (forward primer: 5’-AATATCGTAGCGCCGGGGTA-3′; reverse primer: 5’-GCCTCCTCATCCTCTTCATC-3′) and sequencing by Paisenor Gene Biotechnology Co., Ltd. (Qingdao, China). The positive recombinant *L. lactis* were named MG1363/pMG36e-*p30-*His, MG1363/pMG36e-*p54-*His, MG1363/pMG36e-*p72-*His, MG1363/pMG36e-*p30*-LTB-His, MG1363/pMG36e-*p54*-LTB-His, MG1363/pMG36e-*p72*-LTB-His, and the negative control was named MG1363/pMG36e.

### 2.4. Western blot analysis

The recombinant bacteria were cultured in 2 ml GM17 liquid medium containing Erm (5 μg/ml) at 30°C for 18 h, subsequently transferred to 30 ml GM17 liquid medium with 2% inoculum and incubated at 30°C for 14 h until OD_600_ = 0.8, centrifuged for 10 min at 12,000 rpm at 4°C, and resuspended in pre-chilled PBS. After blending, samples were processed by ultrasonic fragmentation and analyzed by the same quantity in the precipitations of each sample was isolated by sodium dodecyl sulfate 15% polyacrylamide gel electrophoresis (SDS-PAGE). Ultimately, Western blot analysis followed by an anti-His tag monoclonal antibody as the primary antibody and HRP-labeled goat anti-mouse IgG as the secondary antibody (Beijing Zhongshan Jinqiao Biotechnology Co., Ltd.).

### 2.5. Immunization

A total of 40 female New Zealand rabbits were randomly divided into four groups, with 10 rabbits per group (*n* = 10), namely, a PBS group, a MG1363/pMG36e group, a group without LTB adjuvant vaccine, and a group with LTB adjuvant vaccine. In group 1, each rabbit received 3 ml of PBS (pH = 7.4); in group 2, each rabbit was given the same dose concentration of 1.0 × 10^8^ CFU/ml MG1363/pMG36e; in group 3, each rabbit was immunized with the same dose and concentration of 1 ml each of recombinant *L. lactis* MG1363/pMG36e-*p30* + *p54* + *p72*-His, as well as in group 4, 1 ml of each of the recombinant *L. lactis* MG1363/pMG36e-*p30* + *p54* + *p72*-LTB-His immunized rabbits, respectively. Rabbits were immunized on days 0 and 17. Two rounds of immunization were administered at 14-day intervals, each lasting 3 days. The experimental period was 34 days, and the details of the vaccination are described in Table 1.

**Table 1:** Immunization details for rabbits.

Group	Primary (0 day)/Secondary (17 days) immunization	Dose/Concentration	Number of per-group collection samples
1	PBS	3 ml/pH = 7.4	5
2	MG1363/pMG36e	3 ml/1.0 × 10^8^ CFU/ml	5
3	MG1363/pMG36e-p30 + p54 + p72-His	1 ml of each/1.0 × 10^8^ CFU/ml	5
4	MG1363/pMG36e-p30 + p54 + p72-LTB-His	1 mLof each/1.0 × 10^8^ CFU/ml	5

### 2.6. Collection of samples

**Figure 1:**
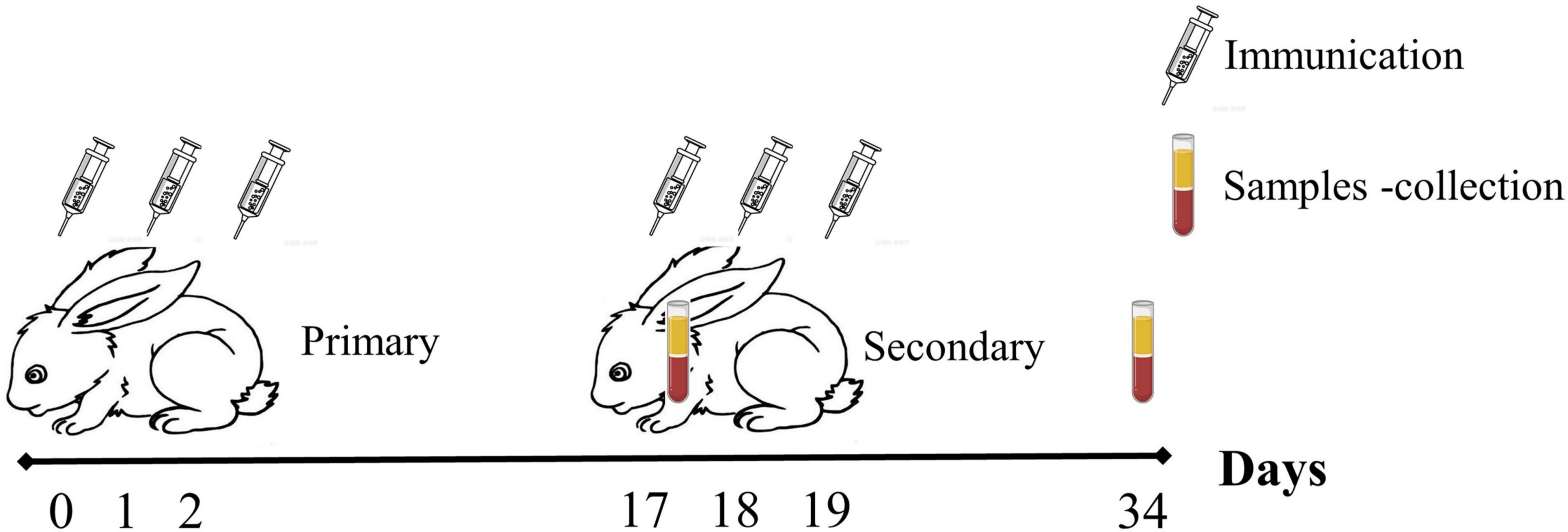
Samples collection of immunizated rabbits.

The samples of the sera, the small intestine for the jejunal segment, and the spleens were collected by five rabbits per group randomly selected on 17th and 34th days post-vaccination (dpv; Figure 1). The blood supernatant was stored at-80°C to access the humoral immune response after centrifuging at 3,000 rpm for 15 min at ambient temperature. Using the same centrifugation methods, treated supernatants from a 5 cm jejunal segment were placed in 5 ml PBS (pH = 7.4) and incubated at 4°C for 2 h to assess mucosal immune response. The collected spleen was rinsed with PBS (pH = 7.4), put in a stainless steel mesh filter with a pore size of 0.075 mm, and ground with a syringe wick until it became white flocculent. The filter was then rinsed and filled with PBS, and the cell suspension was collected in a sterile centrifuge tube and centrifuged for 10 min at 1,500 rpm. The cells were rinsed once with RPMI-1640 culture media and again with PBS. The cells were then suspended in RPMI-1640 culture medium, which contains 10% fetal bovine serum and 100 U m/L of penicillin and streptomycin. Typan blue was then used to demonstrate the viability of the cells, and the cell survival ratio was >90%. After adjusting the cell density, the cells were inoculated at a density of 2 × 10^6^ cells m/L and incubated at 37°C in a 5% CO_2_ incubator, and the samples were stored at –80°C for evaluating the spleen cell survival rate.

### 2.7. Enzyme-linked Immunosorbent assay

The levels of IgG in serum and sIgA in small intestinal contents were determined using the rabbit-derived ASFV p30-IgG, p54-IgG, and p72-IgG enzyme-linked immunosorbent assay (ELISA) kits and the rabbit-derived sIgA ELISA kit (Shanghai Yongwin Biotechnology Co., Ltd.), according to the manufacturer’s instructions. Briefly, the negative control well contained 50 μl, the positive control well contained 50 μl, and the sample wells contained 10 μl of the sample to be tested and 40 μl of diluent, which were added with 100 μl HRP-labeled detection antigen and incubated at 37°C for 1 h. Then washed five times with wash buffer. Subsequently, each well added 50 μl each of substrate A and substrate B which were incubated for 15 min at 37°C, avoiding light. Within 15 min, add 50 μl of termination solution to each well, and after that, the absorbance was read at 450 nm using an enzyme-labeling measuring instrument (Thermo Fisher Scientific, United States). Ultimately, the IgG or sIgA antibody levels of our samples were calculated based on the standard curve of the kits.

### 2.8. Cytokine-release assay

The levels of IL-4 and IFN-γ in serum were performed using ELISA kits according to the manufacturer’s recommendation (Shanghai Landon Biotechnology Co., Ltd.). The method was similar to that previously mentioned in 2.7. The concentrations of IL-4 and IFN-γ in serum were obtained from the standard curve of each ELISA plate.

### 2.9. Spleen cell survival rate assay

The spleen cells from immunized rabbits were prepared for the lymphocyte survival rate assay using the CCK-8 Cell Viability Assay Kit (Shanghai Langdon Biotechnology Co., Ltd.) according to the manufacturer’s instructions. In brief, each well with 100 μl of splenocyte suspension was incubated in a 5% CO_2_ incubator (SANYO, Japan) at 37°C for 24 h. Then, the plate was added 100 μl of complete medium, incubating for 48 h in a 37°C, 5% CO_2_ incubator. After that, 10 μl of CCK-8 solution was added to each well and incubated for 3 h in a 37°C, 5% CO_2_ incubator. Subsequently, the absorbance at 450 nm was measured by an enzyme-labeling measuring instrument (Thermo Fisher Scientific, United States). Finally, Cell viability (%) = (OD_450_ of sample cells/OD_450_ of control cells) × 100%.

### 2.10. Statistical analyzes

The experimental data were analyzed by GraphPad Prism software (San Diego, CA, USA), and the significance of differences between groups was tested by One-way ANOVA.

## 3. Results

### 3.1. Construction of plasmids and recombinant *Lactobacillus lactis*

**Figure 2:**
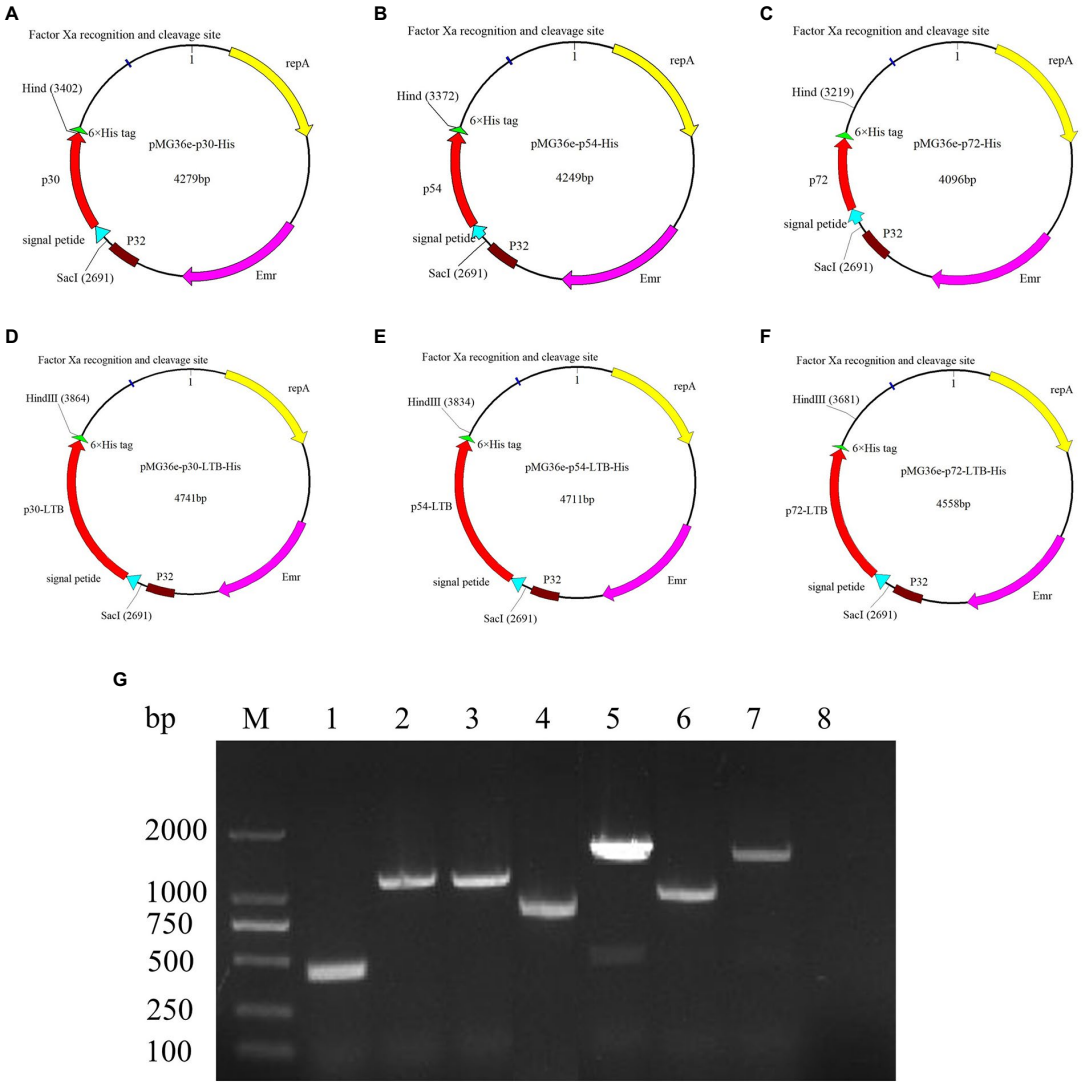
Construction and identification of the recombinant *L. lactis*. **(A)** A map of the plasmid pMG36e-p30-His, the plasmid size is 4,279 bp; **(B)** A map of the plasmid pMG36e-p54-His, the plasmid size is 4,249 bp; **(C)** A map of the plasmid pMG36e-p72-His, the plasmid size is 4,096 bp; **(D)** A map of the plasmid pMG36e-p30-LTB-His, the plasmid size is 4,741 bp; **(E)** A map of the plasmid pMG36e-p54-LTB-His, the plasmid size is 4,711 bp; **(F)** A map of the plasmid pMG36e-p72-LTB-His, the plasmid size is 4,558 bp. **(G)** Identification results of recombinant *L. lactis* by colony PCR, MG1363/pMG36e (lane 1); MG1363/pMG36e-*p30*-His (lane 2); MG1363/pMG36e-*p54*-His (lane 3); MG1363/pMG36e-*p72*-His (lane 4); MG1363/pMG36e-*p30*-LTB-His (lane 5); MG1363/pMG36e-*p54*-LTB-His (lane 6); MG1363/pMG36e-*p72*-LTB-His (lane 7); Negative control (lane 8).

As shown in Figures 2A–F, six new functional recombinant plasmids were successfully constructed. According to Figure 2G, the empty vector and six recombinant *L. lactis* were identified by PCR, and the target bands of recombinant *L. lactis* MG1363/pMG36e, MG1363/pMG36e-*p30*-His, MG1363/pMG36e-*p54*-His, MG1363/pMG36e-*p72*-His, MG1363/pMG36e-*p30*-LTB-His, MG1363/pMG36e-*p54*-LTB-His, MG1363/pMG36e-*p72*-LTB-His were 442 bp, 1,111 bp, 1,081 bp, 928 bp, 1,573 bp, 1,543 bp, and 1,390 bp, respectively, which were consistent with the expected target fragment sizes.

### 3.2. Expression of proteins by recombinant *Lactobacillus lactis*

**Figure 3:**
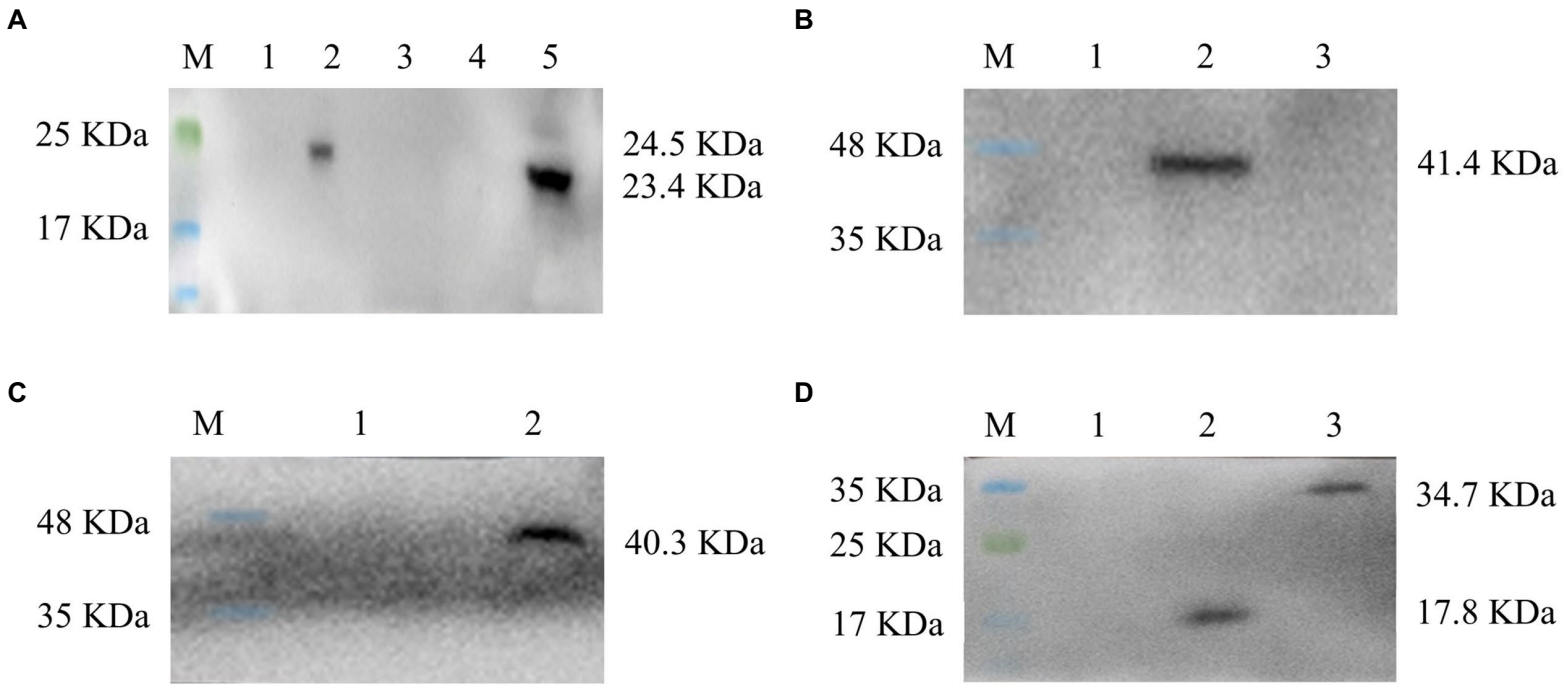
All six constructs, denoted as p30, p54, p72, p30-LTB, p54-LTB, and p72-LTB, were inserted into expression plasmid pMG36e and expressed in *Lactobacillus*. **(A)** The expressions of the recombinant *L. lactis* ASFV p30-His protein and p54-His protein were verified by Western blot, lane 1, 3–4: Negative control, lane 2: p30-His, lane 5: p54-His. **(B)** The expression of the recombinant *L. lactis* ASFV p30-LTB-His fusion protein was verified by Western blot, lane 1: Negative control, lane 2: p30-LTB-His. **(C)** The expression of the recombinant *L. lactis* ASFV p54-LTB fusion protein was verified by Western blot, lane 1–2: Negative control, lane 3: p54-LTB-His. **(D)** The expressions of the recombinant *L. lactis* ASFV p72-His protein and p72-LTB-His fusion protein were verified by Western blot, lane 1: Negative control, lane2: p72-His, lane 3: p72-LTB-His.

The expressions of the p30-His protein, p54-His protein, p72-His protein, p30-LTB-His fusion protein, p54-LTB-His fusion protein, and p72-LTB-His fusion protein were successfully detected by Western blot (with His-tag as the detection antigen) in Figures 3A–D. The p30*-*His and p54*-*His molecular weights were 24.5 kD and 23.4 kD, respectively (Figure 3A). The p30*-*LTB-His molecular weight was 41.4 kD (Figure 3B). The p54*-*LTB-His molecular weight was 40.3 kD (Figure 3C). The p72*-*His and p72*-*LTB-His molecular weights were 17.8 kD and 34.7 kD, respectively (Figure 3D). The results indicated that the proteins of interest were consistent with the expected size, proving that the recombinant *L. lactis* successfully expressed the foreign protein.

### 3.3. Determination of anti-ASFV specific IgG levels in sera

On 17 and 34 dpv, specific antibodies to p30, p54, and p72 were detected in the serum of rabbits. Tables 2–4 demonstrated that there were 4 positive samples in groups 3 and 4 on 17 dpv, with an 80% positive sample rate. On 34 dpv, however, there were 5 positive samples in groups 3 and 4, with a 100% positive rate for the samples. Additionally, on 17 and 34 dpv, neither group 1 nor group 2 had a positive rate. Although this test is qualitative, its methodology allows it to be semi-quantitative, and the size of the OD_450_ value somewhat predicts the level of antibodies. The differences between groups 3 and 4 and groups 1 and 2 are highlighted in the data, suggesting that the recombinant bacteria can induce a humoral immune response.

**Table 2:** The levels of p30 IgG antibody in serum of rabbits.

Sampling time	Group	OD450 (Critical value OD450 = 0.246)					Positive number	Total sample	Positive rate (%)
On 17 dpv	1	0.191	0.238	0.211	0.162	0.102	0	5	0
	2	0.089	0.237	0.222	0.114	0.235	0	5	0
	3	0.386	0.087	0.320	0.370	0.431	4	5	80
	4	0.313	0.131	0.307	0.344	0.513	4	5	80
On 34 dpv	1	0.162	0.153	0.130	0.205	0.141	0	5	0
	2	0.206	0.106	0.092	0.116	0.219	0	5	0
	3	0.471	0.431	0.659	0.456	0.631	5	5	100
	4	0.500	0.661	0.516	0.513	0.525	5	5	100

**Table 3:** The levels of p54 IgG antibody in serum of rabbits.

Sampling time	Group	OD450 (Critical value OD450 = 0.216)					Positive number	Total sample	Positive rate (%)
On 17 dpv	1	0.172	0.137	0.163	0.166	0.158	0	5	0
	2	0.192	0.084	0.172	0.120	0.108	0	5	0
	3	0.298	0.148	0.268	0.286	0.384	4	5	80
	4	0.334	0.191	0.316	0.282	0.310	4	5	80
On 34 dpv	1	0.113	0.177	0.098	0.160	0.140	0	5	0
	2	0.205	0.114	0.097	0.112	0.160	0	5	0
	3	0.372	0.397	0.443	0.381	0.288	5	5	100
	4	0.665	0.529	0.458	0.515	0.441	5	5	100

**Table 4:** The levels of p72 IgG antibody in serum of rabbits.

Sampling time	Group	OD450 (Critical value OD450 = 0.232)					Positive number	Total sample	Positive rate (%)
On 17 dpv	1	0.217	0.162	0.220	0.124	0.187	0	5	0
	2	0.116	0.177	0.187	0.183	0.137	0	5	0
	3	0.318	0.105	0.299	0.354	0.315	4	5	80
	4	0.307	0.134	0.321	0.332	0.340	4	5	80
On 34 dpv	1	0.127	0.219	0.194	0.138	0.128	0	5	0
	2	0.173	0.197	0.093	0.139	0.169	0	5	0
	3	0.621	0.521	0.660	0.499	0.601	5	5	100
	4	0.423	0.330	0.393	0.396	0.371	5	5	100

The results were judged as follows: critical value = negative control hole OD_450_ value +0.15, sample OD value < critical value is judged as negative; sample OD value > critical value is judged as positive.

The OD_450_ of the negative control well of p30 IgG was 0.096, and its critical value OD_450_ = 0.246.

The OD_450_ of the negative control well of p54 IgG was 0.066, and its critical value OD_450_ = 0.216.

The OD_450_ of the negative control well of p72 IgG was 0.082, and its critical value OD_450_ = 0.232.

### 3.4. Determination of sIgA levels In intestinal mucosa

**Figure 4:**
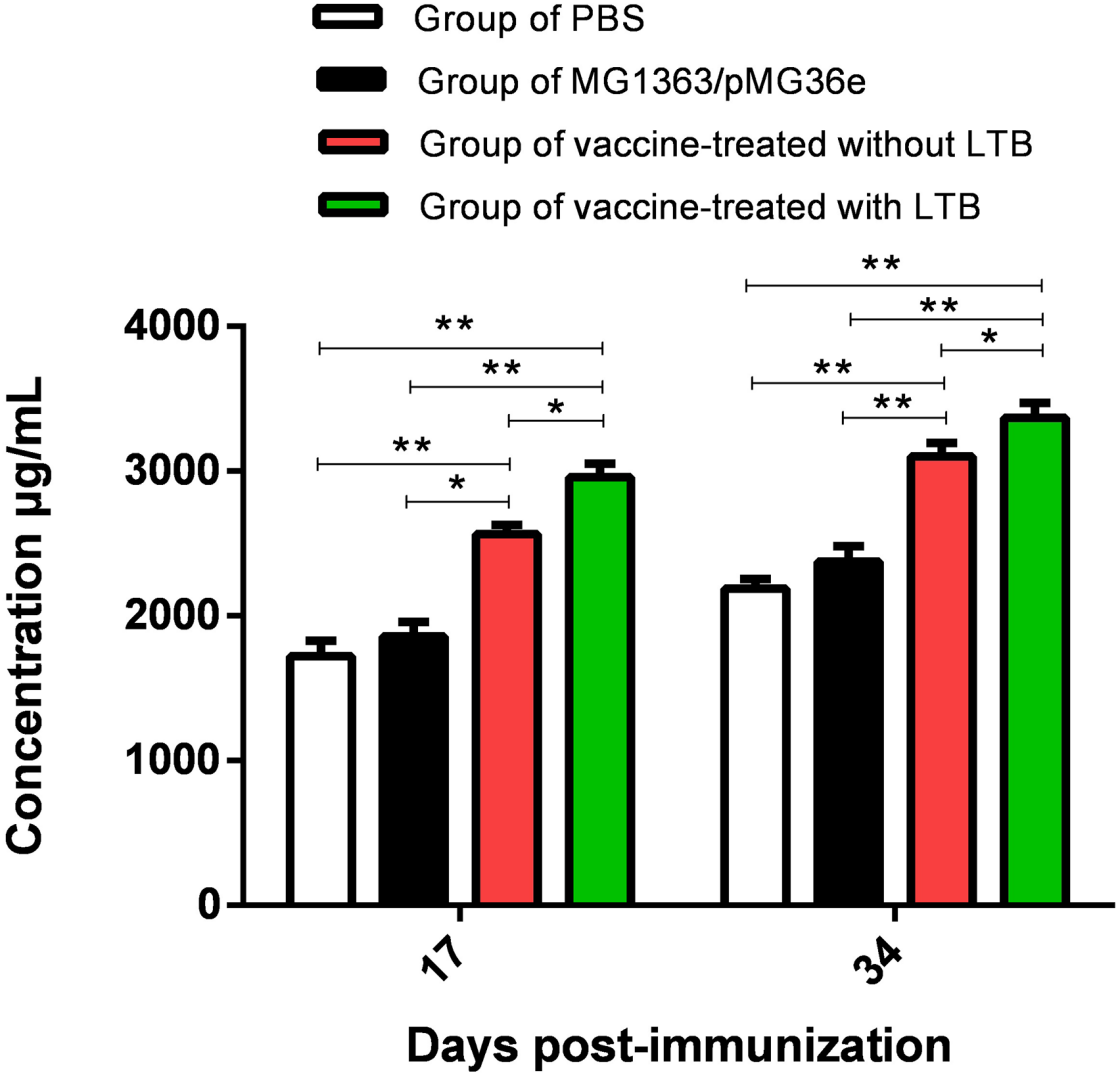
Determination of intestinal mucosa sIgA levels on 17 and 34 dpv by ELISA. “**” in the figure means the difference is extremely significant *p* < 0.01, “*” indicates a significant difference *p* < 0.05, and “ns” indicates no significant difference *p* > 0.05.

The sIgA levels in the intestinal mucosa were measured by ELISA on 17 and 34 dpv. According to the findings in Figure 4, the results showed that the sIgA of groups 3 and 4 were significantly higher than that of groups 1 (*p* < 0.01) and 2 (*p* < 0.01), respectively. Group 4 differed slightly from group 3 (*p* < 0.05), with an increasing trend from days 17 to 34. These data suggested that oral administration of recombinant bacteria could stimulate the mucosal immune system in rabbits.

### 3.5. Determination of cytokines in sera

**Figure 5:**
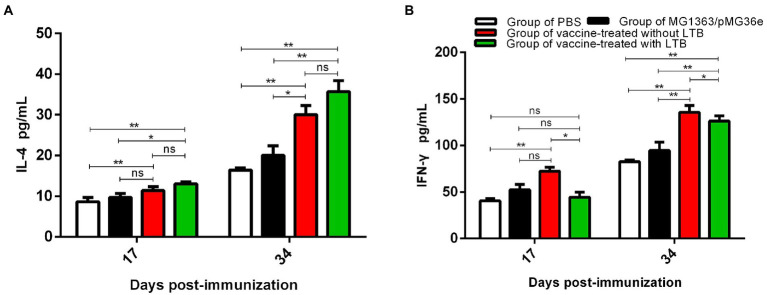
The levels of cytokines IL-4 and IFN-γ in serum were measured using ELISA on 17 and 34 dpv. **(A)** The levels of cytokines IL-4 in sera on 17 and 34 dpv were determined using ELISA. **(B)** The levels of cytokines IFN-γ in sera on 17 and 34 dpv were measured *via* ELISA. “**” in the figure means the difference is extremely significant *p* < 0.01, “*” indicates a significant difference *p* < 0.05, and “ns” indicates no significant difference *p* > 0.05.

The levels of cytokines IL-4 and IFN-γ in serum were measured using ELISA on 17 and 34 dpv (Figures 5A,B). As shown in Figure 5A, the serum IL-4 concentrations of group 3 and group 4 on 17 and 34 dpv were significantly different than those of group 1 (*p* < 0.01), respectively. There was no significant difference (*p* > 0.05) in the level of IL-4 concentrations observed between groups 2 and 3 on 17 dpv, but they were significantly higher (*p* < 0.05) on 34 dpv. However, group 4 performed slightly better (*p* < 0.05) on 17 dpv and significantly better (*p* < 0.01) on 34 dpv than group 2. Although there was no difference (*p* > 0.05), there was an increasing trend between groups 3 and 4. These results demonstrated that vaccination with LTB adjuvant can produce significantly higher levels of Th2-associated cytokine IL-4 in rabbits.

The level of cytokine IFN-γ in serum was determined *via* ELISA on 17 and 34 dpv. The results, as shown in Figure 5B, indicated that on 17 dpv, there was a significant difference between groups 1 and 3 (*p* < 0.01), while no significant differences were found between group 1 and group 4 (*p* > 0.05), and compared to group 2, group 3 and group 4 also showed no difference (*p* > 0.05). Unexpectedly, the level of IFN-γ in group 3 was significantly higher than in group 4 (*p* < 0.05). Following the strength of immunization, the data on 34 dpv suggested that groups 3 and 4 were monumentally higher than groups 1 (*p* < 0.01) and 2 (*p* < 0.01). There was a significant difference between groups 3 and 4 (*p* < 0.05). The results showed that oral administration without the LTB adjuvant resulted in significantly higher levels of the Th1-associated cytokine IFN-γ.

### 3.6. Determination of splenocyte viability

**Figure 6:**
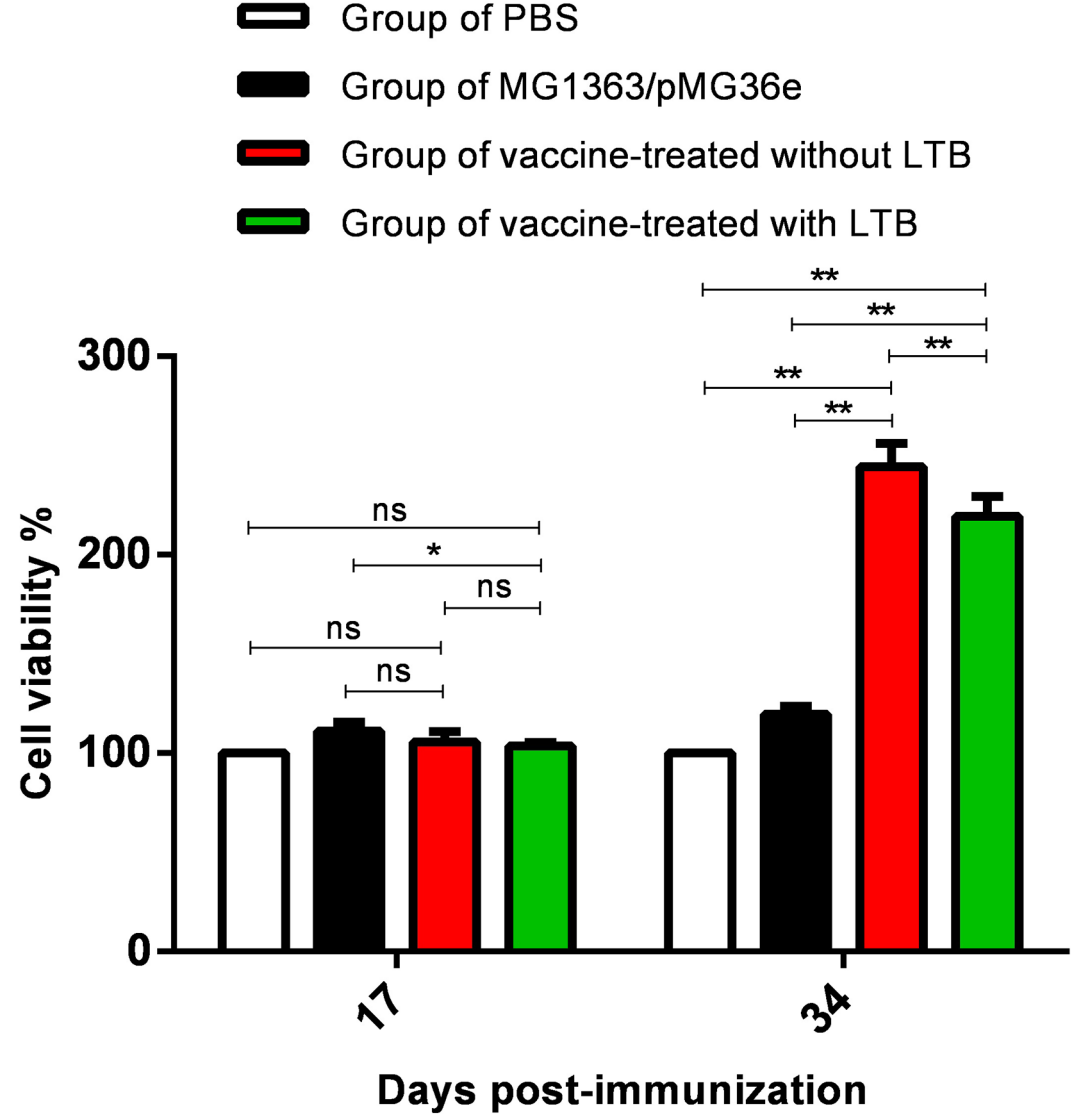
The cell viability of splenocytes after the first immunization on 17 and 34 dpv by CCK-8. “**” in the figure means the difference is extremely significant *p* < 0.01, “*” indicates a significant difference *p* < 0.05, and “ns” indicates no significant difference *p* > 0.05.

The cell viability of splenocytes after the experiment was detected by the CCK-8 kit on 17 and 34 dpv. Only groups 2 and 4 (*p* < 0.05) showed significance in Figure 6 on 17 dpv. On 34 dpv, groups 3 and 4 were monumentally higher than groups 1 (*p* < 0.01) and 2 (*p* < 0.01), respectively, and there was a statistical difference between groups 3 and 4 (*p* < 0.01). These data demonstrated that immunization of rabbits with recombinant bacteria increased the percentage of splenocytes, indicating the activation of T cells in the rabbit model.

## 4. Discussion

Over the past years, large-scale epidemics and outbreaks in swine caused by ASFV have occurred in China and Central Europe, resulting in serious threats to the international pig industry and its environment (Zhou et al., 2018; Wen et al., 2019; Zhao et al., 2019). The key to controlling virus diffusion is an effective vaccine, especially focused on the subunit vaccine of ASFV. Barasona et al. (2019) is the first to report that oral immunization of wild boar with attenuated ASFV of genotype II isolated in Latvia in 2017 provided 92% protection against a challenge with a virulent ASFV isolate, Arm07, highlighting the promising possibility of mucosal-associated immunity against virus infection. Additionally, a review has shown that vaccines administered *via* the oral route can elicit mucosal IgA antibodies, which are necessary for ASF vaccines in the wild boar population (Urbano and Ferreira, 2022). Therefore, developing an ASF subunit vaccine based on mucosal immunity that can overcome limitations on vaccine administration and further develop rapidly adaptable is exigent and imperative (Teklue et al., 2020).

*Lactobacillus* has been shown in studies to colonize the intestine, stimulate the gut for an extended period, and act as a mucosal adjuvant and antigen delivery system (Bermúdez-Humarán et al., 2011). The researchers demonstrated that using *L. lactis* as a host bacterium for *H. pylori* CagL antigen, they were able to detect specific antibodies (IgA and IgG) as well as cytokines (IL-17 and IFN-γ) *via* oral immunization (Aliramaei et al., 2019). A significant discovery was proved that constructed recombinant bacteria and developed an oral vaccine based on *L. lactis* with good prophylaxis against brucellosis (Rezaei et al., 2020). In this study, the experimental group of rabbits had considerably higher serum-specific IgG antibody and small intestinal mucosal sIgA antibody levels, enhancing the trend of antibody levels in the host with the boosting of immunizations, which was consistent with the expected results. Research findings supported our hypothesis that all immunized rabbits developed particular antibodies *in vivo*.

LTB, as an adjuvant with antigen, improves the vaccine’s immune effect, improves cell-mediated immune responses, increases serum IgG and fecal sIgA levels, and plays a role in T cell activation and differentiation (Turcanu et al., 2002; Sun et al., 2013; Peng et al., 2019). The results of this study for serum IgG and small intestinal mucosal sIgA were consistent with previous experiments. Interestingly, the results for cytokines (IL-4, IFN-γ) showed that with LTB adjuvant vaccine group had higher levels of IL-4 than without LTB adjuvant vaccine group, but the results for IFN-γ were the opposite. We hypothesized that LTB could induce Th2 cell immunity while suppressing Th1 cell immunity because it increased TNF-γ and IL-10 production while decreasing IL-12 release and failing to promote Th1 cell development (Donaldson et al., 2011). Similarly, splenocyte survival was inhibited by the LTB adjuvant vaccine group, but the mechanism by which LTB inhibits Th1 cell immunity and splenocyte survival needs to be investigated further.

To date, reliable animal models and biosafety level-3 (BSL-3) laboratories have made it challenging to evaluate the immunological effects of vaccines *in vivo*. A number of recent studies, including those by Lopera-Madrid (Lopera-Madrid et al., 2021), Sugisawa (Sugisawa et al., 2022), Wang (Wang et al., 2022), et al., have used mice as a model to assess ASF experiences. Importantly, promising results by Barasona et al. (2019) provided hope for the design of oral ASFV vaccines. In a similar study, using the *Lactobacillus plantarum* (*L. plantarum*) system to express p14.5 protein *via* the oral route in mice is an ideal method to develop a vaccine strategy (Huang et al., 2022). However, it has been demonstrated that achieving effective protection with a single antigen is difficult. Based on that, using rabbits in our study as a model may provide a fresh perspective, and constructing recombinant *L. lactis* containing three antigens and a fused LTB adjuvant may provide a novel approach to developing an oral ASFV subunit vaccine.

According to the results, oral administration of recombinant *L. lactis* significantly increased rabbits’ resistance, including their humoral, cellular, and mucosal immune systems. We attempt a novel method for inducing a mucosal immune response against ASFV to prevent ASFV infection through mucosal epithelial cells. Next, we will study the protective effect of recombinant bacteria against an ASFV challenge in piglets by constructing more ASFV proteins *via* oral immunization.

## Data availability statement

The original contributions presented in the study are included in the article/Supplementary material, further inquiries can be directed to the corresponding authors.

## Ethics statement

The animal study was performed in the Qingdao Haihua Bio-pharmaceutical Technology Co., Ltd. (Haihua), and accordance with the protocols approved by the Animal Care and Ethics Committee of the Haihua, under the number AUP-QY-C-S(1)-2021–019.

## Author contributions

XC and HS conceived the idea. HoZ and SZ did most of the experimental work and wrote the first draft of the manuscript. HaZ, YS, and PZ collected all the data. HoZ and XC advised in the process of manuscript writing. All authors contributed to the article and approved the submitted version. All authors agreed to be accountable for the content of the work.

## Funding

This work is based upon research funded by Shandong Provincial Major Project of the New-Old Kinetic Energy Conversion [no. (2020)1220], Shandong Provincial Key Research and Development Program (Major Scientific and Technological Innovation; no. 2020CXGC010801-02), Shandong Province agricultural major application technology innovation project: (no. SD2019XM003), and Project ZR2020MC185 supported by Shandong Provincial Natural Science Foundation.

## Conflict of interest

The authors declare that the research was conducted in the absence of any commercial or financial relationships that could be construed as a potential conflict of interest.

## Publisher’s note

All claims expressed in this article are solely those of the authors and do not necessarily represent those of their affiliated organizations, or those of the publisher, the editors and the reviewers. Any product that may be evaluated in this article, or claim that may be made by its manufacturer, is not guaranteed or endorsed by the publisher.
