# Corrigendum: Orally administered recombinant *Lactobacillus* expressing African swine fever virus antigens that induced immunity responses

**DOI:** 10.3389/fmicb.2024.1529166

**Published:** 2024-12-03

**Authors:** Hongliang Zhang, Saisai Zhao, Haojie Zhang, Yu Shen, Peijun Zhang, Hu Shan, Xiulei Cai

**Affiliations:** ^1^College of Veterinary Medicine, Qingdao Agricultural University, Qingdao, Shandong, China; ^2^College of Animal Science and Technology, Shandong Agricultural University, Tai'an, Shandong, China

**Keywords:** African swine fever virus, enterotoxin B subunit, *Lactococcus lactis*, recombinant expression, oral immunization, immunogenicity evaluation

In the published article, there was an error in the Funding statement. The last funding number is incorrect. The correct Funding statement appears below.

